# Evolutionary demography of age at last birth: integrating approaches from human behavioural ecology and cultural evolution

**DOI:** 10.1098/rstb.2017.0060

**Published:** 2018-02-12

**Authors:** Siobhan Mattison, Christina Moya, Adam Reynolds, Mary C. Towner

**Affiliations:** 1 Anthropology, University of New Mexico, Albuquerque, NM 87131, USA; 2 Anthropology, University of California at Davis, Davis, CA 95616, USA; 3 Integrative Biology, Oklahoma State University, 501 Life Sciences West, Stillwater, OK 74074, USA

**Keywords:** stopping behaviour, reproductive cessation, kinship, fertility behaviour, China, matriliny

## Abstract

Cultural evolutionary theory and human behavioural ecology offer different, but compatible approaches to understanding human demographic behaviour. For much of their 30 history, these approaches have been deployed in parallel, with few explicit attempts to integrate them empirically. In this paper, we test hypotheses drawn from both approaches to explore how reproductive behaviour responds to cultural changes among Mosuo agriculturalists of China. Specifically, we focus on how age at last birth (ALB) varies in association with temporal shifts in fertility policies, spatial variation and kinship ecologies. We interpret temporal declines in ALB as plausibly consistent with demographic front-loading of reproduction in light of fertility constraints and later ages at last birth in matrilineal populations relative to patrilineal ones as consistent with greater household cooperation for reproductive purposes in the former. We find little evidence suggesting specific transmission pathways for the spread of norms regulating ALB, but emphasize that the rapid pace of change strongly suggests that learning processes were involved in the general decline in ALB over time. The different predictions of models we employ belie their considerable overlap and the potential for a synthetic approach to generate more refined tests of evolutionary hypotheses of demographic behaviour.

This article is part of the theme issue ‘Bridging cultural gaps: interdisciplinary studies in human cultural evolution’.

## 1. Introduction

Humans reproduce far less than their physiological capacity allows [1]. One indicator of this tendency is a cross-culturally common practice of women stopping reproduction well before they reach menopause [2,3]. Such behaviours appear puzzling from evolutionary perspectives that anticipate women making full use of their reproductive careers to maximize fitness. In this paper, we use two evolutionary frameworks—human behavioural ecology (HBE) and cultural evolutionary theory (CET)—to explore the timing of reproductive cessation among the Mosuo of Southwest China. As discussed in §4, the Mosuo have been officially constrained to a maximum of three children per woman since the implementation of the Chinese fertility policy in the late 1970s. They also consist of two distinct subpopulations—one matrilineal and duolocal and one patrilineal and patrilocal—that reside in distinct geographical areas with very different terrains that affect the spread of information among communities. We make use of these differences (i.e. the fertility policy and differences in kinship ecologies) as quasi-natural experiments [4] to perform simultaneous tests of hypotheses drawn from HBE and CET frameworks.

Just as there are no singular evolutionary predictions, there are no singular behavioural ecological or cultural evolutionary predictions for a given phenomenon, including age at last birth (ALB). Rather, several behavioural ecological and cultural evolutionary models with diverse assumptions suggest different predictions. Human behavioural ecologists emphasize the concept of trade-offs [5] and strategic negotiations [6] in their hypothesis building. CET has traditionally focused hypothesis building on the dynamics of change and the ways that individuals acquire information from others.

The compatibilities and disjunctures between evolutionary frameworks, including HBE and CET, have been debated [7–10], but there is increasing interest in integrating these frameworks [8,11]. The most common empirical attempts at integration (e.g. [12–14]) are arguably in line with Tinbergen's [15] call to establish comprehensive understandings of behaviour, by exploring both proximate (mechanism and ontogeny) and ultimate (evolutionary history and current adaptive value) explanations for a given trait. In this type of integration, HBE may be viewed as seeking ‘ultimate’ explanations for an adaptively plastic trait, whereas CET explanations that focus on social transmission may be viewed as ‘proximate’ mechanisms describing how traits are spread [10]. The approach is integrative in the sense that it recognizes that these answers are necessarily mutually compatible as answers at one level ‘cannot be regarded as also answering another’ [8, p. 716], while accommodating the fundamental interest in HBE of identifying the adaptive value of a behaviour in its current environment [16] versus the focus on how behaviours are spread that is central to CET [17].

This type of integration falls short of truly synthetic approaches envisioned by recent proponents of a ‘new’, or extended, evolutionary synthesis. One of these proponents' main goals is to investigate the ways that other inheritance systems, such as cultural ones, affect evolutionary dynamics and adaptation [8,9]. Such approaches point out that processes other than natural selection can lead to non-genetic adaptations [17]. This is because cultural traits or institutions, somewhat analogously to genes, can be differentially successful at replicating if they enhance their hosts’ survival or reproduction, or if they are preferentially copied. The simplest example of cultural adaptations are tools, such as arrowheads, that evolve to become increasingly adept at performing a function in a given environment. Institutions can be thought of in a similar way if those that provide individual or group benefits in a given environment are more likely to be adopted or persist (e.g. [18,19]). This pushes cultural processes into the domain of ultimate explanation. Complex coevolutionary models between genes and cultural traits and adaptive lags in quickly changing environments arguably dissolve the neat distinction between ultimate and proximate explanations for various phenomena [10,20].

In our view, the synthetic value of this approach applies most clearly to understanding the *dynamics* of evolutionary processes and less clearly affects inferences drawn about the current adaptive value of a trait as it is measured at one place and time (see also [21]). For this project, we lack the intergenerational data necessary to explore the dynamic interactions between transmission and long-term adaptive behaviour. With cross-sectional data from women of varying ages who were subject to the changing institutional landscapes of twentieth century China, we examine (i) how reproductive behaviour changes in response to cultural shifts, (ii) some possible mechanisms of such change and (iii) whether such behaviours are consistent with specific adaptive models of decision-making. While we cannot accomplish an ‘extended’ synthesis, we recognize the importance of multiple levels of causality for the observed variation in ALB. Simultaneous consideration of HBE and CET frameworks is thus, on the one hand, pragmatic [8]; at the same time, it represents a true step forward in attempting to explore both the current utility and the transmission dynamics of ALB strategies.

While we tried to devise comprehensive hypotheses sets before seeing the data, our analysis is largely exploratory. We review several hypotheses drawn from HBE and CET to posit explanations for the pathways influencing spatial and temporal variation in the timing of ALB. We focus on a population of ethnic Mosuo in Southwest China where fertility has been officially restricted for nearly 40 years. This allows us to examine reproductive *timing* decisions in a context where they are unlikely to reflect downstream effects of decisions made with respect to total fertility. Furthermore, the context affords the opportunity to study the impacts of (i) top–down institutional policy changes such as the ‘one child policy’ on temporal variation, (ii) matri- versus patri-focal kinship institutions on spatial variation and (iii) educational variation on both spatial and temporal variation in individuals' strategies towards the end of their reproductive careers.

## 2. Age at last birth in evolutionary perspective

Several evolutionary models used by human behavioural ecologists suggest possible adaptive motivations for earlier reproductive stopping. Such models have close ties to related phenomena, including fertility decline and the evolution of menopause and a long post-reproductive lifespan. First, earlier reproductive stopping may be used to manage quality/quantity trade-offs when intermediate levels of reproductive output maximize fitness [22,23]. The decision to terminate reproduction at an earlier age could be consistent with a quality-focused strategy if it allowed parents to invest more intensively in existing offspring, particularly while their energetic reserves are relatively high [24]. On the other hand, *later* ALB could also be construed as an investment in offspring quality if this were associated with increased time-sensitive parental inputs (e.g. breastfeeding) into offspring who are more widely spaced, or higher-quality parenting after accumulation of greater social or economic capital. Particularly in the former case, we would anticipate a corresponding shift to lower lifetime reproductive output and improved indicators of child quality (e.g. child health). Mattison *et al.* [25] have argued previously that earlier reproduction is associated with increased availability of potential allocarers among the matrilineal Mosuo. If so, we might anticipate that the presence of allocarers favours *later* ALB, reflecting a longer reproductive span with no trade-off in child quality.

In the absence of strong quality–quantity trade-offs, a more potent consideration in evolutionary models concerns the effects of demography on reproductive timing. Earlier reproduction is favoured in stationary and growing populations. This idea is encapsulated by Fisher's [26] concept of reproductive value [5]: earlier-born offspring represent a greater marginal benefit to parental reproductive success (RS) than later-born offspring and are also favoured, given sufficient spacing between offspring, due to future discounting [5]. This is because earlier bouts of reproduction shorten generation times and earlier-born individuals constitute a higher relative proportion of the population gene pool [27–29]. Delayed reproduction also increases the likelihood that a woman will die before the birth or maturation of her next child [30]. Timing is thus often as important a determinant of fitness as the total reproductive output, which is only a true reflection of fitness in stationary populations [5]. Thus, in a society where total fertility is tightly regulated, but where the population was still growing, as was the case after the implementation of various fertility restriction policies in China, we anticipate earlier timing of births being beneficial with respect to fitness.

A third class of evolutionary game theoretic models focuses on reproductive negotiations between members of a household. Sometimes termed ‘cooperative breeding models’, various accounts posit that the cost of reproduction may be alleviated by the availability of allocarers (e.g. maternal grandmothers or older daughters) who may promote continued reproduction [31–33]. There is some empirical support for the importance of older daughters' contributions to their mothers' reproductive success and prolonged reproductive careers. For example, Bereczkei & Dunbar [34] showed later ALB for Roma women who had first-born daughters as compared to those with first-born sons and interpreted this as evidence of first-born daughters acting as ‘helpers at the nest’. Similarly, Turke [35] found higher ALB and lifetime RS for families with first-born daughters among the matrilocal Ifaluk of Melanesia.

However, within cooperative systems, there is often conflict and competition over household resources and the recipients of allocare [3,36], which could also bear on timing of ALB. Cant & Johnstone [36] have argued that women who recently married patrilocally, and are thus genetically unrelated to their marital households, have a higher stake in the competition over who gets to reproduce than do their mothers-in-law, who by later life would be related to many household members. Thus, ALB is predicted to be earlier in patrilocal contexts because mothers-in-law cede the competition to their daughters-in-law. In matrilocal contexts, by contrast, a woman would be as related to any offspring she produced as to those produced by her parents, which would result in the conflict being resolved in favour of a woman's mother, thus favouring later ALBs. Snopkowski *et al*. [3] did not find evidence to support these predictions when they compared ALB and the age of menopause between matrilocal and patrilocal women in Indonesia. Stronger evidence for a conflict model of reproductive cessation was found in a study of pre-industrial Finns, where intergenerational overlap in reproduction led to decreased offspring survivorship [37]. In the Chinese context, we might anticipate such reproductive conflicts, and therefore the associations between ALB and residence strategy being stronger before reductions in fertility reduced the potential for reproductive overlap.

The above models are agnostic as to the ways that people achieve these equilibria or fitness-enhancing strategies. While individual learning, evolved psychological mechanisms and physiological responses may play roles, it is likely that cultural learning contributes to the spread of strategies about timing of ALB. This is because (i) learning what is optimal reproductive timing in a vast range of socio-ecological conditions is a difficult task that does not lend itself to trial-and-error learning and (ii) the moralization of reproductive behaviours is cross-culturally pervasive [38,39] and requires learning local norms in order to coordinate with others. Several empirical studies have found evidence for social transmission affecting reproductive behaviours. While Alvergne *et al.* [13] found that social transmission of contraception norms was less important than individual factors such as parity and education in explaining the uptake of contraception in Ethiopia, education itself may show an effect because it is an avenue for social learning. Colleran and co-workers [12] find that social influences were important both with respect to the uptake of contraception and in relation to the specific form of contraception that women chose to use in rural Poland. This study also reinforced the importance of community-level characteristics (e.g. mean level of education) in affecting the dynamics of social transmission. Howard & Gibson [14] also show that female genital cutting (FGC) in West Africa plausibly persists due to mechanisms of cultural transmission and that the adaptive value of FGC appears higher in contexts where the practice is more common, suggesting the importance of coordinating norms. Several models have suggested that the increased importance of educational systems with teachers who act as cultural models and have lower fertility may be implicated in fertility declines. Empirical evidence supports the importance of both group- [12] and individual-level [40] education in fertility decline. This pattern is consistent with accounts that stress social models (such as teachers) with lower fertility behaviours serving as vectors of new norms and with accounts that stress the new economic and reproductive trade-offs for individually educated women. To our knowledge, pathways illuminating how ALB is spread have not previously been explored. In this paper, we inspect patterns of spatial clustering across villages and temporal change in ALB to draw inferences about the role of social transmission in the timing of ALB among the Mosuo.

## 3. Hypotheses

Before we test more specific hypotheses derived from HBE and CET frameworks, we examine whether there is spatial (across villages) and temporal (across cohorts) variation in ALB. There are many reasons why ALB might be structured at the village level. This could be due to village-level norms surrounding reproductive strategies specifically (e.g. regarding the timing of births or the length of reproductive careers), or other norms that have downstream effects on reproduction (e.g. education is important, so reproductive careers should be pushed back). Villages could also differ in their ecologies (e.g. terrain ruggedness) in ways that would affect reproductive timing (e.g. because women engage in more strenuous labour in some villages, which prevents them from conceiving as easily at older ages). The most obvious reason for temporal variation in ALB in this context is the top–down implementation of the Chinese fertility policy in the late 1970s that restricted fertility to a maximum of three children for ethnic minorities such as the Mosuo living in rural areas [41]. The *wan-xi-shao* (late–long–few) messaging of the earlier part of the decade, by which the government encouraged voluntary later births, longer birth intervals and fewer children, resulted in lower fertility in China [42,43] and might be associated with shifts in ALB. Even before these national policies, in the 1940s and 1950s, the Mosuo exhibited lower fertility than their neighbours [44], suggesting that local reproductive norm differences may have already been in play. Furthermore, global trends associated with the demographic transition, including increasing importance of education, are likely to play a role in any cohort changes we might find [45].

All of these cultural and institutional shifts changed the fitness landscapes for different reproductive strategies among the Mosuo. Possible implications of different findings are outlined below in H1–H5.

H1. Temporal variation in ALB across the twentieth century will be partly explained by the late–long–few messages of the 1970s, and the so-called one-child policy, which began being implemented in 1979. This can produce various patterns.

(a) Top–down policies to reduce fertility may result in earlier ALB if earlier reproduction is favoured [25] as parents move to reproduce earlier in order to front-load reproduction. Furthermore, people ‘caught’ in the middle of their reproductive careers when policies were enacted might have earlier last births just by virtue of having met fertility quota.
(b) If fertility policies served as a cue that population sizes were likely to start decreasing, people may have pursued later fertility schedules, as later ALB is hypothesized to be advantageous in shrinking populations [5].
(c) Bottom–up shifts in fertility may have preceded policy implementation [43,46]; if so, then there may be no clear association between policy implementation and ALB.

H2. Spatial variation in village-level ALB will be partly explained by kinship systems. If reproductive conflict between women in stable reproductive unions is a determinant of female ALB, we expect earlier ALB in patrilocal communities.

H3. Education both changes life-history trade-offs and introduces new norms.

(a) If the higher embodied capital and norms associated with education mean pushing reproductive careers later, we would expect a positive association between education and ALB, controlling for fertility.
(b) Insofar as education accounts for the historical shifts associated with demographic transitions, we expect cohort effects to drop out once we account for education.

H4. Differences seen in ALB might be by-products of other changes in reproductive decisions that shift in response to policies or local socio-ecology.

(a) If ALB changes as a consequence of total fertility, we expect the effects of cohort, education and village-level variance to attenuate once we account for fertility.
(b) If ALB changes as a consequence of a later shift in reproductive career, we expect AFB to show similar cohort effects to ALB.

Finally, patterns of spatial and temporal heterogeneity can also speak to how ALB and related norms are culturally transmitted and spread. Villages in the matrilineal region are in less rugged and more accessible terrain than those in the patrilineal region. Therefore we might expect that:

H5. Information spreads more quickly in matrilineal than patrilineal regions and in villages close to the major market town. This may be manifested as:

(a) greater homogeneity in ALB in matrilineal regions or
(b) faster changes in ALB in matrilineal regions and in villages nearer to the market town.

## 4. Study population and data collection

The data for this study were collected over nine months in 2008 in both patrilineal and matrilineal communities of the ethnic Mosuo of Southwest China. The Mosuo are one of 55 officially recognized ethnic minorities in China [47] now numbering over 40 000 [40] and residing on the border of Sichuan and Yunnan Provinces surrounding the picturesque Lugu Lake. The matrilineal subpopulation of the Mosuo are best known to social scientists: this subpopulation resides in the flatlands of the Hengduan Mountains, practises matrilineal descent, in which lineage membership is conferred via females [48] and inheritance, in which resources are passed on from senior generations of matrilineally related individuals to all junior members of the household [49]. Under typical circumstances, only descendants of household females would be present to inherit, as husbands and wives normatively maintain separate residences (i.e. they are duolocal), practising non-committal reproductive unions known as ‘walking marriages’ [50], whereby children that result from these unions reside with their mothers. The patrilineal Mosuo reside in distinct but neighbouring geographical regions, in steeper areas of the Hengduan Mountains. Although they share much in common with their matrilineal counterparts, including language, attire, certain customs, religious beliefs and even blood relations, they differ almost entirely in their systems of inheritance, descent and marriage [51]. Descent and inheritance are patrilineally reckoned, marriage is monogamous, and postmarital residence normatively patrilocal. The terrain is also especially rugged, movement between villages correspondingly difficult, and access to the market town quite limited. Land is more circumscribed for individual households due to this rugged terrain [52]. The patrilineal Mosuo are, on the aggregate, poorer than the matrilineal Mosuo, whose wealth is also more variable; two Mosuo villages at the time the study was deployed were particularly wealthy due to the influence of tourism (see also [53]). Wealth differences increased beginning in the 1980s, when tourism become an important source of income and probably affected a relatively small fraction of Mosuo families prior to that. Our test of the conflict model of timing in ALB is thus anchored in a setting where the major distinctions in subpopulations are based on kinship, and possibly ecology, as opposed to broader normative distinctions in cultural ideologies.

By contrast, the Chinese fertility policy provides a relatively clean test of how a pinpointed change in the cultural regulation of reproduction may have affected ALB. Neither subpopulation of the Mosuo could be considered to display natural fertility [54]. Barrier contraception is used commonly to regulate the timing of childbearing, as is tubal ligation when no further children are desired. Since *ca.* 1979, the Mosuo have been subject to the Chinese fertility policy. As a minority population, they were allowed a maximum of three children at the time of our surveys. Although the implementation of the fertility policy was variable across China [55], it left a clear signature among the Mosuo, with declines in fertility evident beginning with cohorts born after 1950 [56,57]. The implementation of this policy thus provides a ‘natural experiment’ [4] through which to investigate the effects of rapid cultural transitions on reproductive behaviour.

To do so, we examine the demographic records of women drawn from 12 villages in both patrilineal (*N* = 5 villages) and matrilineal (*N* = 7 villages) subpopulations of the ethnic Mosuo. These records were obtained via direct interviewing of a single respondent in each of 228 households. Interviews were administered in Mandarin Chinese or in the local dialect by a member of the research team, or translated into Naru, the Mosuo language, by a local assistant [52]. Each respondent was asked to provide information on all individuals who had been born in the household, even if they resided elsewhere at the time the survey was given. In these analyses, we focus on a subset of data pertaining to stopping reproduction.

## 5. Data and analyses

To study ALB, we examine a sample of reproductive women from the 2008 household surveys (*n* = 320 women). This sample includes all resident women who were at least 30 years old, who had had one or more children and who had complete data on the modelled variables. Variables include ALB—our dependent variable—and the following covariates: age; cohort; education; normative lineality of community of residence (matrilineal versus patrilineal) and village of residence; distance to market town; fertility (number of surviving children); and age at first birth (AFB); see table 1 for summary statistics and detailed descriptions.

**Table 1.** Descriptive statistics of sample population, broken down by lineality.

variable name		matrilineal	patrilineal	combined	variable description
		**mean** **(s.d.)**	**mean** **(s.d.)**	**mean** **(s.d.)**	
age		48.6 (14.8)	47.7 (13.4)	48.4 (14.6)	approximate age at time of interview
fertility		2.8 (1.7)	2.6 (1.1)	2.8 (1.6)	number of live births
AFB		23.1 (4.1)	21.0 (2.8)	22.7 (4.0)	age minus oldest child's age
ALB		29.2 (6.4)	26.3 (4.6)	28.7 (6.2)	age minus youngest child's age
distance		8.1 (7.3)	48.8 (2.7)	15.2 (16.9)	village-level distance (km) to primary market town
		***N* (%)**	***N* (%)**	**total**	
lineality		264 (82.5)	56 (17.5)	320	residence in normatively patrilineal or matrilineal area
cohort	<1955	34 (94.4)	2 (5.6)	36	5-year intervals centred on calendar year at age 16
	1955–1959	15 (71.4)	6 (28.6)	21	
	1960–1964	16 (76.2)	5 (23.8)	21	
	1965–1969	23 (85.2)	4 (14.8)	27	
	1970–1974	18 (81.8)	4 (18.2)	22	
	1975–1979	19 (76.0)	6 (24.0)	25	
	1980–1984	41 (83.7)	8 (16.3)	49	
	1985–1989	54 (81.8)	12 (18.2)	66	
	1990–1994	44 (83.0)	9 (17.0)	53	
education	none	207 (82.1)	45 (17.9)	252	level of highest school attended
	elementary	36 (80.0)	9 (20.0)	45	
	middle+	21 (91.3)	2 (8.7)	23	
village					village of current residence (*N* = 12)

To examine temporal variation, we use age to construct a reproductive cohort variable based on 5-year intervals. This cohort variable is centred to show the period during which the woman was 16 years of age, corresponding to the earliest AFB in the data. Compared with linear age, the cohort variable allows us to detect nonlinear changes over time, including those that the various fertility policies of the 1970s might have precipitated.

To examine spatial variation, we use multilevel models with random effects for the specific village in which a woman resided. As described above, villages are located in normatively matrilineal and patrilineal subpopulations, which are spatially clustered. Lineality thus captures both the kinship system and a larger region. The distance variable gives geodesic distance in kilometres between each specific village and the main market town.

We evaluate our predictions through estimation and comparison of multilevel survival statistical models. We define as right-censored all women between 30 and 44 who gave birth within the past 6 years, irrespective of the number of children. Given demographic trends [58], this is a conservative censoring rule—many censored women would likely have had their final child. All statistical analyses are conducted in R [59]. To use both survival and mixed modelling approaches, we use the *coxme* package to estimate cox proportional hazard models with village as a random effect, allowing the intercept to vary [60]. We evaluate the resulting models using a model comparison approach based on information criteria [61,62]. AIC differences are used to calculate model weights among different subsets of models according to the hypothesis being evaluated. The weight of the model is a function of the distance (difference) in AIC values between models and is a measure of the relative likelihood that it is the best model given the data among the models being compared [63].

## 6. Results

For coherence, we report results in the order that we presented our hypotheses.

**Figure 1.**
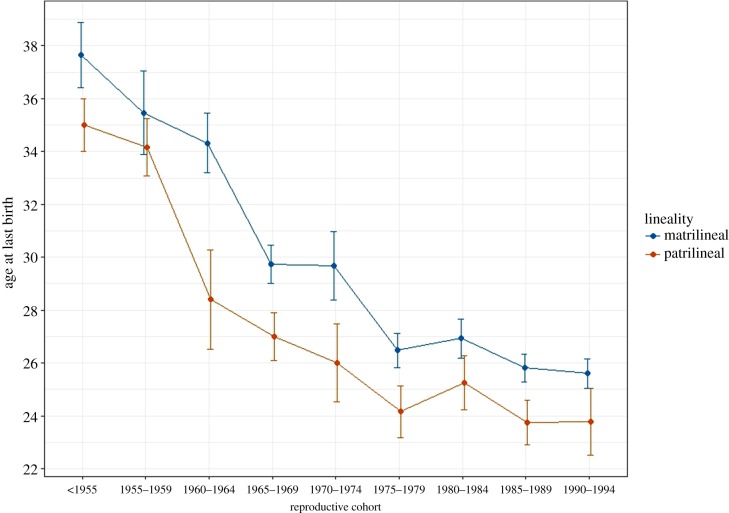
Age at last birth (ALB) by cohort. ALB shows declines in mean and median age and in variability across cohorts in our sample. Note that the steepest decline occurs between the cohort of women reaching maturity prior to 1960 and the following cohorts. The first cohort includes the most women who would have finished reproduction by the enactment of the Chinese fertility policy.

H1. ALB exhibits a downward temporal trend that is apparent across the full sample of women (figure 1). The model with reproductive cohort receives much stronger support (*w* = 1) than a model without cohort, despite the jump in the number of estimated parameters (table 2; ‘cohort’). Older women in the sample—particularly those who reached 16 years old before 1960—have later ALBs than younger women, as well as greater variation in ALB; i.e. younger cohorts are progressing faster to their last births (table 1). That said, the changes in ALB clearly began prior to the late, long, few campaign of the 1970s and the one-child policy of 1979. Although ALB continues to drop for women whose prime reproductive years occur during these policies, there is no clear evidence of a time threshold—i.e. of an abrupt change in ALB that maps onto new fertility policies.

**Table 2.** Model estimates predicting progression to last birth. Column values are exponentiated coefficients for the variables in the given model, followed by 95% confidence intervals in brackets. In simple terms, exponentiating the coefficients gives us a description of how, for a particular woman's realized values for the independent variables, the risk of having an event (e.g. a last birth) increases or decreases relative to the comparison group. For ALB, a value greater than 1 increases the likelihood (risk) of a last birth relative to the base risk, whereas a value less than 1 decreases the likelihood of a last birth relative to the base risk [60]. AIC, Akaike information criterion.

		cohort	cohort + lineality	cohort + fertility	cohort + education	cohort + distance	full
cohort	(<1955)						
	1955–1959	1.46 [0.83, 2.54]	1.35 [0.77, 2.36]	1.49 [0.85, 2.60]	1.47 [0.84, 2.56]	1.35 [0.77, 2.36]	1.40 [0.80, 2.45]
	1960–1964	2.03 [1.15, 3.59]	1.94 [1.11, 3.42]	2.23 [1.26, 3.95]	1.99 [1.12, 3.52]	1.96 [1.11, 3.46]	2.07 [1.17, 3.66]
	1965–1969	3.39 [1.96, 5.87]	3.43 [1.99, 5.93]	2.76 [1.59, 4.78]	3.38 [1.95, 5.85]	3.37 [1.95, 5.83]	2.74 [1.58, 4.75]
	1970–1974	3.19 [1.80, 5.65]	2.96 [1.70, 5.17]	2.44 [1.37, 4.34]	3.16 [1.78, 5.59]	3.04 [1.73, 5.34]	2.27 [1.28, 4.02]
	1975–1979	6.39 [3.63, 11.28]	6.12 [3.47, 10.80]	4.42 [2.47, 7.92]	6.69 [3.77, 11.85]	6.19 [3.51, 10.91]	4.42 [2.46, 7.96]
	1980–1984	4.85 [2.97, 7.92]	4.47 [2.77, 7.22]	3.10 [1.84, 5.23]	4.99 [3.05, 8.18]	4.52 [2.79, 7.33]	2.98 [1.77, 5.01]
	1985–1989	4.78 [2.95, 7.72]	4.44 [2.77, 7.12]	2.80 [1.64, 4.78]	5.53 [3.36, 9.11]	4.52 [2.81, 7.26]	3.02 [1.75, 5.20]
	1990–1994	2.09 [1.20, 3.64]	2.00 [1.15, 3.47]	1.22 [0.67, 2.23]	2.41 [1.36, 4.27]	1.98 [1.14, 3.45]	1.31 [0.71, 2.43]
lineality	(matrilineal)						
	patrilineal		1.95 [1.40, 2.73]				1.86 [1.28, 2.70]
fertility				0.82 [0.74, 0.91]			0.82 [0.74, 0.91]
education	(none)						
	elementary				1.17 [0.82, 1.68]		1.28 [0.89, 1.83]
	middle+				0.40 [0.21, 0.76]		0.43 [0.23, 0.81]
distance						1.02 [1.01, 1.02]	
village-level variance		0.092	0.008	0.097	0.080	0.021	0.023
negative log likelihood		−1321.25	−1315.67	−1314.28	−1315.43	−1316.28	−1303.616
d.f. (*k*)		9	10	10	11	10	13
AIC_i_		2660.5	2651.4	2648.5	2652.9	2652.6	2633.232
AIC_i_ − AIC_cohort_		0	−9.1	−12.0	−7.6	−7.9	−27.3

H2. We do not find evidence of structured village-level variation when village is entered as a random effect in models of ALB (table 2). That is, a model with the random effect is not an improvement over a null model with no random effect; likelihood ratio = 0.144. Despite this, there is a large effect of a village's kinship system on ALB, with women from patrilineal villages being more likely to experience an earlier ALB (table 1 and figure 1). The model that includes lineality in addition to cohort is strongly favoured (*w* = 0.99) over a model with cohort only (table 2; ‘cohort + lineality’).

H3. Including education also leads to a much better model (*w* = 0.98) than the cohort-only model, with women with the highest levels of education being more likely to have a later ALB (table 2; ‘cohort + educaton’). Variation in education does not, however, account for the initial temporal change across cohorts in ALB given the relatively small number of women in the higher education category and the fact that they are limited to more recent reproductive cohorts. Nevertheless, the model suggests that the highest educational levels might indeed delay reproductive cessation, countervailing other temporal influences; this trend is nonlinear, with women at intermediate (elementary) levels of education actually progressing faster to a last birth.

H4. Including fertility in the temporal model of ALB leads to an improved model (*w* = 1), but does not completely attenuate the cohort effects (table 2; ‘cohort + fertility’). This suggests that there were some historical shifts in decision-making about ALB that were independent of fertility reduction goals. Rather than include AFB directly into a model of ALB (due to collinearity for women with just one child), we modelled AFB itself using the same approach as we do for ALB and found no support for a sustained temporal shift in AFB. This suggests that the effects of ALB are not being driven by changes in AFB (electronic supplementary material, table S2).

H5. There is little evidence that ALB variation is structured in ways that reflect recent cultural transmission events in the region. We find that a model with distance to market town does receive some support (*w* = 0.35) when compared with the lineality (*w* = 0.64) and temporal models (*w* = 0.01). That said, the distance to market is confounded by the patrilineal and matrilineal regions, because the patrilineal villages are all farther from town. Moreover, the matrilineal villages show less change over time in ALB, counter to the predicted direction if norms were flowing from the market town to the nearer smaller villages. Finally, the matrilineal villages are not more homogeneous than the patrilineal ones, counter to the prediction of an easier flow of information in the less rugged matrilineal areas (electronic supplementary material, figure S1).

## 7. Discussion

Timing of reproductive cessation among humans is highly variable. Significant effort in the evolutionary sciences has been expended to explain this variation as it arises physiologically, i.e. in terms of the timing of menopause (e.g. [3,24,64]), with explicit interest in the facultative (i.e. behavioural) adjustment of timing of ALB arising more recently (e.g. [2]). In this paper, we test several hypotheses explaining variation in the distribution of ALB drawn from HBE and CET frameworks.

In particular, we show that ALB varies across time among the Mosuo, with women from earlier cohorts reproducing until later ages. The drops in ALB are fairly consistent through time, casting doubt on a primary causal role of 1970s Chinese fertility policies in motivating different timing decisions. Such temporal shifts in the timing of reproduction plausibly reflect increasing orientation towards front-loading of reproduction in response to third-party regulation of total fertility behaviour in growing populations. Second, we show that almost all spatial variation in ALB is accounted for by villages being in the matrilineal versus patrilineal areas. Specifically, ALB is later among women residing in matrilineal areas. Third, we show that variation in education does not account for cohort effects, while shifts in fertility and AFB only partially account for the temporal shifts in ALB. This suggests that shifts in embodied capital investments for mothers or investments in child quality are unlikely explanations for ALB variation. Finally, we find no evidence that ALB-relevant norms spread geographically—neither proximity to cities nor terrain ruggedness associated with the patrilineal areas was associated with ALB homogeneity or the rate of ALB change.

The timescale at which we see changes to the ALB suggests that cultural (rather than genetic or strictly ecological) shifts have influenced reproductive decision-making among the Mosuo. Mean ALB decreases 10 or more years within two decades (figure 1). Furthermore, much of this shift takes place before the Chinese fertility policy was implemented. It is difficult to interpret why this earlier decline occurred. On the one hand, this may imply that both bottom–up and top–down cultural forces influenced late-life reproductive timing if Mosuo villagers were already moving towards anti-natalist norms [44]. For example, it may suggest that the Chinese fertility policy was aligned to some degree with already changing reproductive norms [43,46]. On the other hand, the Chinese Communist Party (CCP) was well established in this region by 1953 and had begun regulating reproductive behaviour among the Mosuo by 1958, when they required Mosuo families to abandon non-committal reproductive unions in favour of monogamous marriage [44]. The Great Leap Forward (1958–1962) resulted in widespread famine and depressed fertility all over China (e.g. [65]) that may have had effects on the timing of ALB in our sample of Mosuo women. Notably, these policies regulated fertility, marriage and subsistence. We know of no specific sanctions against continued reproduction at advanced ages among the Mosuo, so the specific mechanisms of social transmission remain obscure. Nonetheless, the regional scale at which we see village-level differences in ALB suggests that cultural norms associated with matri- or patrilineal institutions may play a role in reproductive timing. While the patrilineal area is in more rugged terrain, the fact that ALB has dropped so dramatically as conditions improved in the later twentieth century suggests that people are ceasing to reproduce earlier than physiological constraints influenced by a difficult working environment would mandate. It is thus unlikely that genetic differences or simple ecological differences with physiological knock-on effects can account for the variation in reproductive timing documented here.

The pattern of declining ALB alongside fertility restriction is consistent with behavioural ecological models that envisage benefits to earlier reproduction [5]. While front-loading strategies might be transmitted via cultural learning mechanisms, we find no evidence that ALB behaviour spread out from regional market centres. As argued previously for the Mosuo case [25], earlier reproduction should be especially valuable under circumstances where fertility is constrained but populations are growing. Indeed, top–down policies dramatically curtailing fertility were very likely to have contributed to future discounting as the context for bearing and raising children became increasingly insecure, enhancing the motivation for early reproduction. Even if ALB is not the direct target of such a strategy (i.e. because earlier onset of reproduction alongside fertility reduction could automatically result in earlier ages of last birth), the fitness effects of reducing one's age at childbearing would be present across all births [5,28]. ALB, *per se*, could be targeted as a strategy for investing in children while one is a younger and healthier parent, or as a mechanism of reducing quantity of offspring to invest in their quality (e.g. [24]). As argued in the introduction (§1), even under conditions of severe fertility restriction, early termination of reproduction could leave a mother in better condition (physiologically and financially) to invest in her offspring. Future work could assess this by exploring the condition of children born to mothers who terminate their reproduction early versus late.

One of our more intriguing results provides evidence that is consistent with models of ALB emphasizing the potential for household cooperation and conflict to affect the timing of reproduction. In particular, our models show relatively early ALB among Mosuo residing in patrilineal areas compared with Mosuo residing in matrilineal areas. At face value, this may seem to support the idea that daughters-in-law win reproductive competition with their mothers-in-law in patrilocal households ([36], but see [3]). However, as this society falls on a relatively low end of the fertility spectrum, it is likely to show relatively little intergenerational overlap and therefore relatively little potential for conflict. This suggests the need for alternative explanations of the regional pattern and casts some doubt on interpretations of similar patterns in the broader literature [3,36,37,65]. The fact that educational and fertility differences do not account for the regional differences between the matrilineal and patrilineal areas' ALBs points to other reasons for the observed patterns in ALB. Furthermore, if easier transmission of market-based norms accounted for the difference, we would expect earlier ALB among the more market-integrated matrilineal villages than the patrilineal ones. One possibility is that ageing women expect reproductive conflict in patrilocal areas and moderate their reproductive timing accordingly, but this account would require a rather inflexible mechanism that cannot handle the mismatch to the modern low fertility context. Future research should collect more direct data on intergenerational reproductive overlap, household composition, economic conditions and individual household-level contributions to allow for more direct tests of how either real or perceived household conflict may impact ALB and other fertility behaviours in the context of differing kinship norms.

An alternative explanation for the kinship structure results suggests that matrilineal kin are more cooperative than patrilineal kin, at least insofar as they provide more alloparental care. If this were the case, we might expect to find our current pattern of longer reproductive spans (6.12 versus 5.3 years, on average) and higher fertility among the matrilineal villages than the patrilineal ones (table 1). There is also lower household-level competition for subsistence resources such as land in the matrilineal region, and households are correspondingly larger. In light of this interpretation, it is a bit surprising that we see later ages at *first* birth for matrilineal than patrilineal women. However, this is consistent with older daughters acting as helpers at the nest for longer periods in the matrilineal villages. While the traditional ethnographic accounts of matrilineal Mosuo suggest a high prevalence of half-siblings that would disincentivize such helping at the nest [66], our own data show that mixed paternity sibsets were rare at the time these data were collected [67]. Better evidence on the actual contributions of alloparents and fuller explanations for any kin-structured differences in cooperation await further research.

We have limited ability to speak to how mothers use ALB strategically to allocate resources between reproduction and other goals, or as a way to manage the quality versus quantity of her children. However, we do find that higher fertility is associated with a slower progression to last birth. Relatively early stopping may therefore be part of a fertility reduction strategy. Similarly, women may be shifting their reproductive careers in response to educational investments that they make in themselves. Women with middle school or higher educational levels have the slowest progressions to last births. This interpretation must remain fairly guarded given that women with intermediate educational levels in fact show the fastest progression to last births, meaning that any effects of education on ALB are not linear. Furthermore, the highest levels of education observed in these communities at the time data were collected (i.e. high school) are unlikely to directly interfere with earlier reproductive careers [25]. This means that any effects of education would have to be due to later trade-offs given different levels of embodied capital or shifting norms acquired through, or reflected in, educational institutions. Finally, the fact that historical shifts towards lower fertility and higher maternal education do not completely account for the earlier ALBs in more recent cohorts suggests that reproductive stopping is changing for other reasons. Further data on various kinds of investments in children, the long-term effects of education for adult women and the kinds of social models present in schools would help address the role of education in shifting norms and perceived reproductive trade-offs.

It is worth reiterating that HBE and CET perspectives often do not make divergent predictions insofar as CET proposes mechanisms of transmission whereby equilibria predicted by HBE models are reached. However, in contexts of change, CET perspectives have the potential to offer additional insights regarding the dynamics of change. For example, during these periods of change, it may be possible to examine humans’ reproductive decision-making rules and the extent to which they may deviate from fitness maximization because of changing norms or institutions. This helps us make sense of the otherwise puzzling evidence that early adopters of low fertility norms suffer long-term fitness losses even as their descendants benefit in social status ([68], but see [5]). Extrapolating from the literature on cultural shifts towards fertility reduction suggests that evolved biases in the types of status cues we attend to, coupled with social learning heuristics, may play a role in other reproductive decisions.

The results presented here should be taken with some measure of caution. First, excluding nulliparous women means that women with later ages at first birth (after age 30) are systematically under-represented in the younger age cohorts, possibly masking effects of delayed childbearing on ALB and other reproductive behaviours. Similarly, women who have not given birth in at least 6 years were classified as post-reproductive, which may also underestimate ALB if some of these women plan to continue reproducing. However, the reverse would also be true (some women with completed fertility would be censored). Furthermore, it should be noted that no women reproducing prior to 1979 were censored by these criteria. However, for earlier cohorts, the data may include survivorship biases, which could be problematic if, for example, women who lived longer also had later ALBs. This may be important when evaluating the impacts of the Chinese fertility policy on reproductive behaviours. Given that our results suggest that ALB began declining prior to implementation of the fertility policy, artefacts arising from this censorship or selection biases are likely to be small.

Another challenge for analysis is that cross-sectional demographic data are limited in the degree to which they can characterize cultural transmission of reproductive behaviours. While cultural evolution studies typically emphasize equilibria resulting from dynamic models of social learning processes, we are limited to the use of crude proxies for transmission dynamics such as geographic proximity to market towns and kinship norms (matri-/patri-lineality). Without being able to link transmission mechanisms directly with outcomes, we are unable to interpret patterns in terms of specific social learning mechanisms or their predicted effects on the adoption and spread of reproductive norms. Social network studies might be a slight improvement, because they can identify assortative clustering and frequency-dependent behaviours (e.g. [69]), but more direct measures will be needed in order to parse out the complementary contributions of HBE and CET mechanisms to ALB behaviours and patterns in reproductive behaviours more generally. We suggest that researchers interested in reproductive decision-making directly examine people's beliefs about various trade-offs, which currencies they value, what they consider high-quality traits for their children and how such beliefs are represented in social networks and among potential social models of different statuses. Intergenerational data could also help with theorizing about changing fitness landscapes for different strategies as culture shifts. This could take the form of better long-term fitness indicators and diverse measures of child quality (e.g. health, educational and economic outcomes) that can hint at parents' motivations for lower fertility or earlier reproduction.

## 8. Conclusion

The timing of reproductive events in human life history can have important consequences for individual fitness, a fact that may be increasingly salient as variation in fertility continues to decline worldwide. Given that ALB is often a stronger predictor of reproductive success than more commonly used proxies such as onset of menarche or AFB [2,70], we anticipate increased interest in this topic. ALB, like lifetime fertility, has been showing declines in many populations over time [71,72] and, indeed, may be one of the most important contributions to the reductions in fertility associated with global fertility transition [72,73]. Consistent with this, our data show clear declines in ALB, which seem to have arisen independently of the Chinese fertility policy. Our results also show that the matrilineal areas exhibit later ALB than patrilineal areas, consistent with kinship systems shaping household-level cooperation, and conflict over reproductive behaviour. Given that there is limited reproductive overlap between generations in these communities, cooperation among alloparents in matrilineal areas is a more likely explanation for this pattern. Our results regarding temporal shifts are most consistent with demographic front-loading models that postulate increased marginal returns to early childbearing. Although we find limited evidence that supports any particular pathway directing the social transmission of norms regulating the timing of ALB, we caution that the data were not designed to explore social transmission and advocate for increased integration of data collection efforts and models from different streams of thought in the evolutionary social sciences. As we have demonstrated in this paper, HBE and CET offer distinct but compatible perspectives on the evolution of reproductive behaviours. That ALB appears to change rapidly and is coupled to kinship norms in these communities suggests that stopping behaviours may adapt to cultural shifts as well as socio-ecological incentives.

## Supplementary Material

Supplementary tables and figures.

## Data accessibility

Data and code for analysis from this paper may be accessed via the corresponding author's ResearchGate page: http://bit.ly/2v8POst.
