# Supplementary material for: Evolutionary demography of age at last birth: integrating approaches from human behavioural ecology and cultural evolution
Source: Philos Trans R Soc Lond B Biol Sci. 2018 Feb 12;373(1743):20170060. doi: 10.1098/rstb.2017.0060 (PMC5812972; doi:10.1098/rstb.2017.0060)

**Online Supplementary Material for Mattison et al., [Title]**

**Table S1. Models of the cohort effect within exclusively matrilineal or patrilineal regions, along with an interaction model. See Table 2 legend for interpretation of coefficients.**

|  |  | **Matrilineal (n=264)** | **Patrililneal**  **(n=56)** | **Lineality x Cohort**  **(n=320)** |
| --- | --- | --- | --- | --- |
| **Cohort** | **(<1955)** |  |  |  |
|  | **1955-59** | 1.47 [0.79, 2.73] | 1 [0.19, 5.17] | 1.48 [0.8, 2.74] |
|  | **1960-64** | 1.85 [1, 3.43] | 5.83 [0.89, 38.43] | 1.87 [1.01, 3.46] |
|  | **1965-69** | 3.35 [1.87, 5.99] | 12.35 [1.64, 93.12] | 3.58 [2.01, 6.39] |
|  | **1970-74** | 2.78 [1.53, 5.05] | 14.18 [1.88, 106.9] | 2.81 [1.56, 5.08] |
|  | **1975-79** | 5.77 [3.11, 10.72] | 24.18 [3.41, 171.44] | 6.27 [3.37, 11.63] |
|  | **1980-84** | 4.35 [2.62, 7.23] | 15.37 [2.35, 100.38] | 4.43 [2.69, 7.31] |
|  | **1985-89** | 4.02 [2.42, 6.66] | 24.59 [3.81, 158.68] | 4.05 [2.46, 6.68] |
|  | **1990-94** | 2.1 [1.16, 3.82] | 2.65 [0.45, 15.7] | 2.2 [1.21, 3.98] |
| **Lineality** | **(matrilineal)** |  |  |  |
|  | **patrilineal** |  |  | 2.01 [0.47, 8.53] |
|  |  |  |  |  |
| **Village-level variance** |  | 0.0109 | 0.0004 | 0.0007 |
|  |  |  |  |  |
| **Interaction: Patrilineal** | **(<1955)** |  |  |  |
|  | **1955-59** |  |  | 0.72 [0.13, 3.99] |
|  | **1960-64** |  |  | 1.13 [0.2, 6.57] |
|  | **1965-69** |  |  | 0.8 [0.13, 4.81] |
|  | **1970-74** |  |  | 1.2 [0.2, 7.29] |
|  | **1975-79** |  |  | 0.89 [0.16, 4.94] |
|  | **1980-84** |  |  | 0.92 [0.18, 4.69] |
|  | **1985-89** |  |  | 1.44 [0.3, 7.03] |
|  | **1990-94** |  |  | 0.64 [0.11, 3.65] |
|  |  |  |  |  |
| **negative LL** |  | -1034.01 | -147.24 | -1314.00 |
| **df (k)** |  | 1 | 1 | 18 |

**Table S2. Model of cohort effect on AFB**

|  |  | **Cohort (n=320)** |
| --- | --- | --- |
| **Cohort** | **(<1955)** |  |
|  | **1955-59** | 1.76 [1.01, 3.06] |
|  | **1960-64** | 2.16 [1.24, 3.77] |
|  | **1965-69** | 1.72 [1.03, 2.87] |
|  | **1970-74** | 1.82 [1.06, 3.14] |
|  | **1975-79** | 1.97 [1.16, 3.33] |
|  | **1980-84** | 1.56 [1.00, 2.43] |
|  | **1985-89** | 1.60 [1.05, 2.44] |
|  | **1990-94** | 1.47 [0.95, 2.27] |
| **Village-level variance** |  | 0.062 |
| **negative LL** |  | -1522.2 |
| **df (k)** |  | 9 |

**Table S3. ALB by cohort**

| **Reproductive Cohort** | **ALB Mean** |  | **ALB Median** | **Range** | **SD** | **n total** |
| --- | --- | --- | --- | --- | --- | --- |
| < 1955 | 37.5 |  | 39 | 21 - 51 | 7 | 36 |
| 1955 - 59 | 35.1 |  | 35 | 23 - 47 | 5.3 | 21 |
| 1960 - 64 | 32.9 |  | 34 | 21 - 43 | 5.0 | 21 |
| 1965 - 69 | 29.3 |  | 28 | 25 - 36 | 3.4 | 27 |
| 1970 - 74 | 29 |  | 28 | 22 - 44 | 5.3 | 22 |
| 1975 - 79 | 25.9 |  | 25 | 22 - 33 | 2.9 | 25 |
| 1980 - 84 | 26.7 |  | 26 | 20 - 38 | 4.5 | 49 |
| 1985 - 89 | 25.4 |  | 24 | 20 - 36 | 3.8 | 66 |
| 1990 - 94 | 25.3 |  | 25 | 17 - 33 | 3.7 | 53 |

**Figure S1. ALB by village. Note: villages labeled M# are from the matrilineal region while those labeled P# are from the patrilineal area.**

**
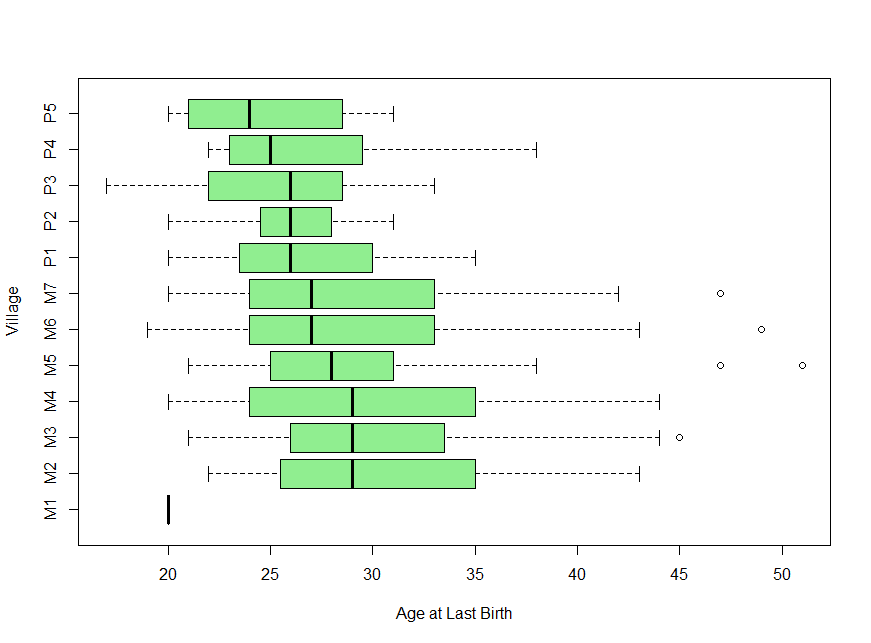
**

**Figure S2. AFB by cohort and lineality**


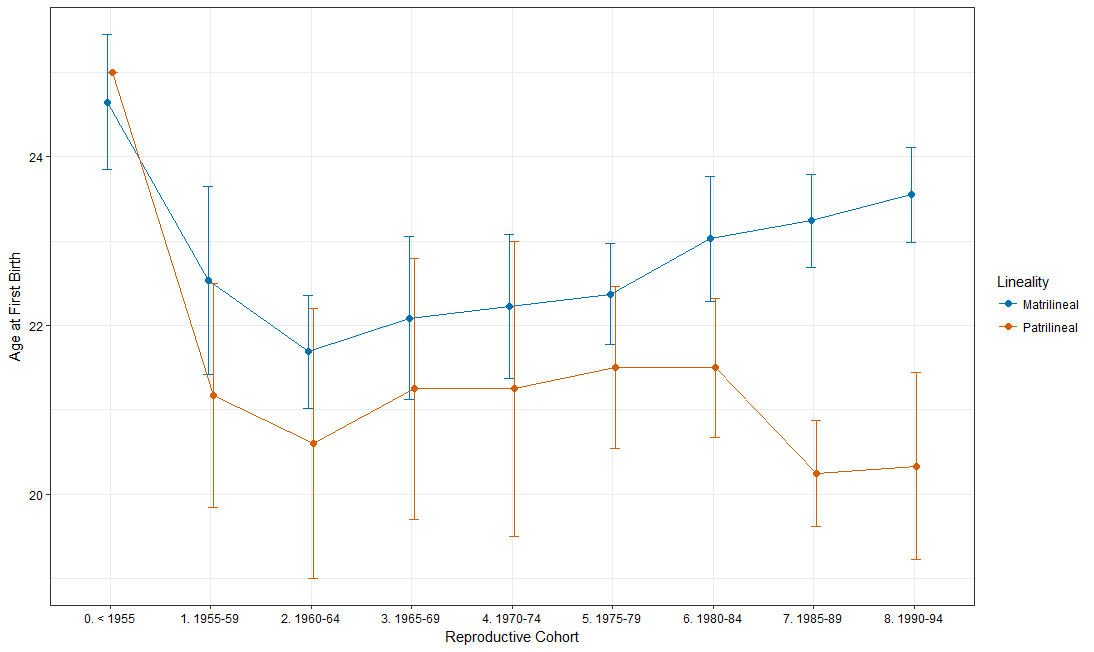


**Figure S3. Fertility by cohort and lineality**


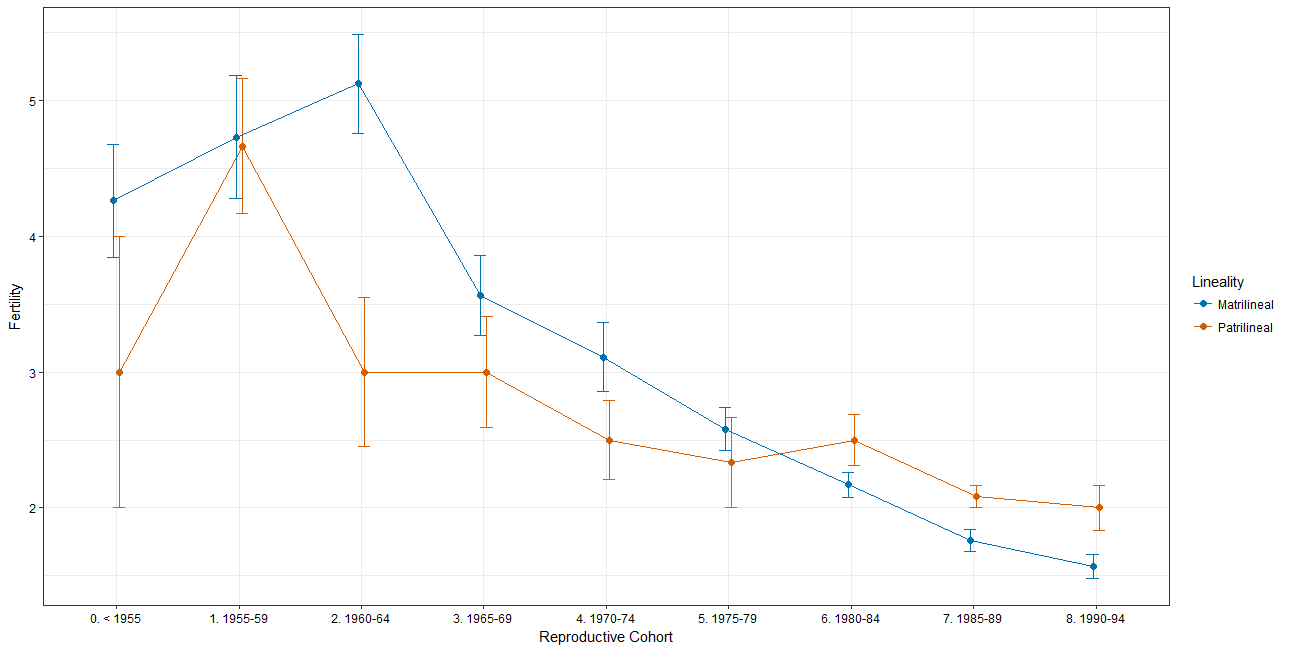

Supplement: Supplementary tables and figures. [file rstb20170060supp1.docx]
